# Molecular characterisation of the *STRUBBELIG-RECEPTOR FAMILY *of genes encoding putative leucine-rich repeat receptor-like kinases in *Arabidopsis thaliana*

**DOI:** 10.1186/1471-2229-7-16

**Published:** 2007-03-30

**Authors:** Banu Eyüboglu, Karen Pfister, Georg Haberer, David Chevalier, Angelika Fuchs, Klaus FX Mayer, Kay Schneitz

**Affiliations:** 1 Plant Developmental Biology, Science Center Weihenstephan, Technical University Munich, Am Hochanger 4, 85354 Freising, Germany; 2 MIPS, Institute for Bioinformatics, GSF National Research Center for Environment and Health, Ingoldstädter Landstrasse 1, 85764 Neuherberg, Germany; 3 Division of Biological Sciences, 304 Life Sciences Center, University of Missouri, Columbia, MO 65211, USA

## Abstract

**Background:**

Receptor-like kinases are a prominent class of surface receptors that regulate many aspects of the plant life cycle. Despite recent advances the function of most receptor-like kinases remains elusive. Therefore, it is paramount to investigate these receptors. The task is complicated by the fact that receptor-like kinases belong to a large monophyletic family with many sub-clades. In general, functional analysis of gene family members by reverse genetics is often obscured by several issues, such as redundancy, subtle or difficult to detect phenotypes in mutants, or by decision problems regarding suitable biological and biochemical assays. Therefore, in many cases additional strategies have to be employed to allow inference of hypotheses regarding gene function.

**Results:**

We approached the function of genes encoding the nine-member STRUBBELIG-RECEPTOR FAMILY (SRF) class of putative leucine-rich repeat receptor-like kinases. Sequence comparisons show overall conservation but also divergence in predicted functional domains among SRF proteins. Interestingly, *SRF1 *undergoes differential splicing. As a result, SRF1 is predicted to exist in a standard receptor configuration and in a membrane-anchored receptor-like version that lacks most of the intracellular domain. Furthermore, *SRF1 *is characterised by a high degree of polymorphism between the L*er *and Col accessions. Two independent T-DNA-based *srf4 *mutants showed smaller leaves while *35S::SRF4 *plants displayed enlarged leaves. This is in addition to the *strubbelig *phenotype which has been described before. Additional single and several key double mutant combinations did not reveal obvious mutant phenotypes. Ectopic expression of several *SRF *genes, using the 35S promoter, resulted in male sterility. To gain possible insights into *SRF *gene function we employed a computational analysis of publicly available microarray data. We performed global expression profiling, coexpression analysis, and an analysis of the enrichment of gene ontology terms among coexpressed genes. The bioinformatic analyses raise the possibility that some *SRF *genes affect different aspects of cell wall biology. The results also indicate that redundancy is a minor aspect of the *SRF *family.

**Conclusion:**

The results provide evidence that *SRF4 *is a positive regulator of leaf size. In addition, they suggest that the *SRF *family is characterised by functional diversity and that some *SRF *genes may function in cell wall biology. They also indicate that complementing reverse genetics with bioinformatical data mining of genome-wide expression data aids in inferring hypotheses on possible functions for members of a gene family.

## Background

Receptor-like kinases (RLKs) constitute a prominent class of receptors that transmit a signal across a membrane. The years since the first isolation of a plant RLK [1] have witnessed a great increase in knowledge regarding the function of plant RLKs. RLKs are required for cellular communication in many processes during the plant's life cycle, regulating aspects of development, defense, and physiology [2-6]. The importance of RLKs in plants is emphasized by the observation that RLKs constitute about 2.5% of the Arabidopsis protein coding sequences [7,8]. Moreover, greater than 200 RLKs belong to the leucine-rich repeat (LRR) class of RLKs typified by varying numbers of LRRs in their extracellular domain. LRRs are involved in protein-protein interactions and are found in numerous types of proteins [9]. Presently, the biological roles of only a handful of RLKs are known. Thus, one important task consists of gaining information about the functions of the remaining plant RLKs.

The functional analysis of genes encoding RLKs is often complicated due to redundancy issues. For example, during early anther development there are repeated requirements for position-dependent intercellular signaling events mediated by discrete sets of redundantly acting LRR-RLKs. The development of the tapetum and the differentiation of microspores depend on the function of several LRR-RLK genes. Analysis of mutations in a single gene, encoding the LRR-X class LRR-RLK EXCESS MICROSPOROCYTES 1/EXTRA SPOROGENOUS CELLS (EXS/EMS) [10,11] revealed a role for *EXS*/*EMS *in these aspects of anther development. Anthers of *exs/ems *mutants fail to form a tapetum and show an increased number of aberrantly developing pollen mother cells. Further, *SOMATIC EMBRYOGENESIS RECEPTOR KINASE 1 *(*SERK1*) and *SERK2 *encode two homologous LRR-RLKs of the LRR-II class [12,13] that are coexpressed during early anther development. While *serk1 *and *serk2 *single mutants both exhibit a wild-type anther morphology, the *serk1 serk2 *double mutants resemble *exs*/*ems *single mutants [12,13].

This finding suggests functionally redundant roles for *SERK1 *and *SERK2 *in processes that also depend on *EXS*/*EMS *function. Interestingly, translational fusions of SERK1 and SERK2 to variants of green fluorescent protein can form homo- and heterodimers in a cell culture system, indicating that SERK1/SERK2 may act in the same protein complex [12]. Another early aspect of anther development is the asymmetric cell division of archesporial cells and the differentiation of primary parietal and primary sporogenous cells. This process is effected by *BARELY ANY MERISTEM *(*BAM1*) and *BAM2 *[14]. *BAM1 *and *BAM2 *are members of a gene family encoding LRR-RLKs, that, in addition to *BAM1 *and *BAM2*, includes *CLAVATA1 *(*CLV1*) and *BAM3 *[15].

The examples described above represent relatively straightforward cases of functional redundancy, where very similar genes are co-expressed in the same tissue and are likely to functionally substitute for each other. Conversely, there are also known instances where a diversification of function has taken place in evolution, largely through alterations in gene expression patterns between closely related LRR-RLK genes. This can result in a combination of partially overlapping and partially separate functions.

For example, signal transduction involving brassinosteroids (BRs) plays an important role in cell elongation and differentiation [16,17]. *BRASSINOSTEROID INSENSITIVE1 *(*BRI1*) encodes a LRR-RLK that constitutes a key component of a BR receptor complex [18-20]. The Arabidopsis genome contains several close relatives of *BRI1*: the *BRI1*-like genes *BRL1*, *BRL3 *and *VASCULAR HIGHWAY1 *(*VH1*)/*BRL2 *[18,21-23]. VH1 does not bind BR but is required for the maintenance of provascular differentiation [21,22]. *BRI1*, *BRL1 *and *BRL3 *encode proteins that share the capacity to bind BR [20,22]. The three genes, however, differ in their expression patterns and there is a shift of emphasis regarding their functions. While *BRI1 *is expressed in a broad fashion, *BRL1 *and *BRL3 *are predominantly expressed in a complementary pattern in vascular tissue [18,20,22-24]. In accordance with its expression pattern, *BRI1 *exerts a broad function in cell elongation and differentiation, which includes the differentiation of vascular tissue. *BRL1 *and *BRL3 *can fully substitute for *BRI1 *[22,23]. However, *BRL1 *and *BRL3 *are mainly required for vascular differentiation, whereby synergistic interactions of *BRI1 *with *BRL1 *and *BRL3 *are required for regular vascular development [22]. A similarly complex behavior is exhibited by the small group of genes encoding ERECTA (ER) and ERECTA-LIKE1 (ERL1) and ERL2 LRR-RLKs that regulate organ size and stomata development [25-27].

The LRR-class of RLKs has been subdivided into several classes (*LRRI*-*LRR-XIII*) [7]. We are interested in the *LRR-V*/*STRUBBELIG-RECEPTOR FAMILY *(*SRF*) gene family encoding putative LRR-RLKs [7]. This monophyletic family is represented by *STRUBBELIG *(*SUB*) and eight additional members. *SUB *was originally identified in a screen for mutants with a defect in ovule development [28]. In more recent work it was shown that *SUB *encodes a putative LRR-RLK of central importance to the plant as it affects cellular morphogenesis in a number of different organs [29]. In particular, *SUB *is required for the orientation of the cell division plane and the control of cell number, cell size and cell shape. Furthermore, *SUB*, also known as *SCRAMBLED *(*SCM*), affects root hair specification [30,31]. A combination of biochemical and genetic evidence suggests that phosphotransfer activity of the kinase domain is not essential for SUB protein function [29]. Thus, SUB is likely to represent an atypical or "dead" RLK [32]. In this paper we report on the initial molecular and functional characterisation of the other members of the *SRF *gene family.

## Results

### The *LRR-V/SRF *gene family encodes putative LRR-RLKs

**Figure 1:**
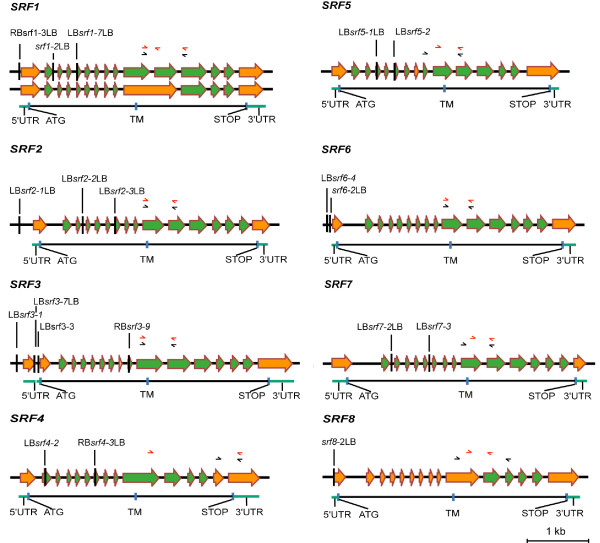
**Molecular organisation of *SRF1-8***. The large arrows indicate exons. Green arrows mark exons that had been correctly predicted while orange arrows highlight corrected exon annotation based on the cDNA and sequence analysis presented in this work. The insertion sites of the various T-DNAs are indicated. The small black arrows represent the primers used to investigate expression of the gene in the indicated homozygous *srf *T-DNA insertion mutants. The small red arrows highlight primers used to investigate differential splicing. Abbreviations: LB, left border of T-DNA; RB, right border of T-DNA; TM, transmembrane domain; UTR, untranslated region.

SUB belongs to LRR-V family of Arabidopsis LRR-RLKs [7]. Further database searches failed to identify additional family members in Arabidopsis. Thus, the LRR-V family encompasses 9 different representatives encoded by genes that are located throughout the genome. We coined the term *STRUBBELIG-RECEPTOR FAMILY *(*SRF*) and named the individual family members *SRF1 *to *SRF8 *[33]. *SUB *would be *SRF9 *but retained its original name (Table 1). *SRF *members also relate to the *ltk *gene family of unknown function from corn [34]. We compared the genomic coordinates of *SRF *genes to investigate whether some pairs are located in segments derived from the youngest large-scale duplication event in Arabidopsis [35]. The pairs *SRF1/SRF3*, *SRF4/SRF5 *and *SRF6/SRF7 *were found as segmentally duplicated pairs while *SUB*, *SRF2 *and *SRF8 *were not located in duplicated regions. We isolated tentative full-length cDNAs of all members (see Methods) to characterize the intron-exon organisation of the *SRF *genes (Figure 1). During this work it became clear that all *SRF *genes carried incorrect annotations in the Arabidopsis database. The reannotation information was submitted to the MIPS Arabidopsis database [36] and to TAIR [37].

**Table 1:** Accession codes of the *SRF *family members

Gene	AGI Code	GenBank Accession
SUB (SRF9)	At1g11130	AF399923
SRF1A (Col)	At2g20850	AY518286
*SRF1B *Col		DQ914918
*SRF1A *L*er*		DQ914919
*SRF1B *L*er*		DQ914920
*SRF2*	At5g06820	AY518287
*SRF3*	At4g03390	AY518288
*SRF4*	At3g13065	AY518289
*SRF5*	At1g78980	AY518290
*SRF6*	At1g53730	AY518291
*SRF7*	At3g14350	AY518292
*SRF8*	At4g22130	AY518293

**Figure 2:**
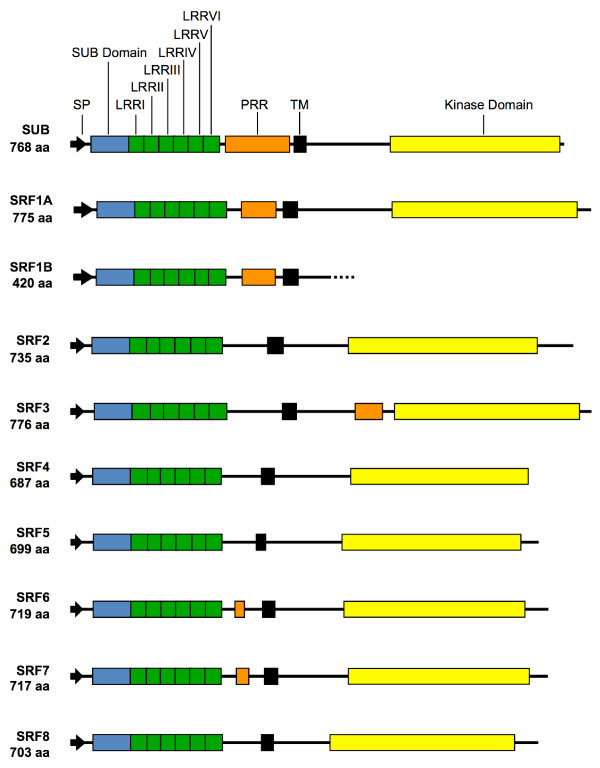
**Domain organisation of SRF proteins**. Abbreviations: LRR, leucine-rich repeat; PRR, proline-rich region; SP, signal peptide; TM, transmembrane domain.

**Figure 3:**
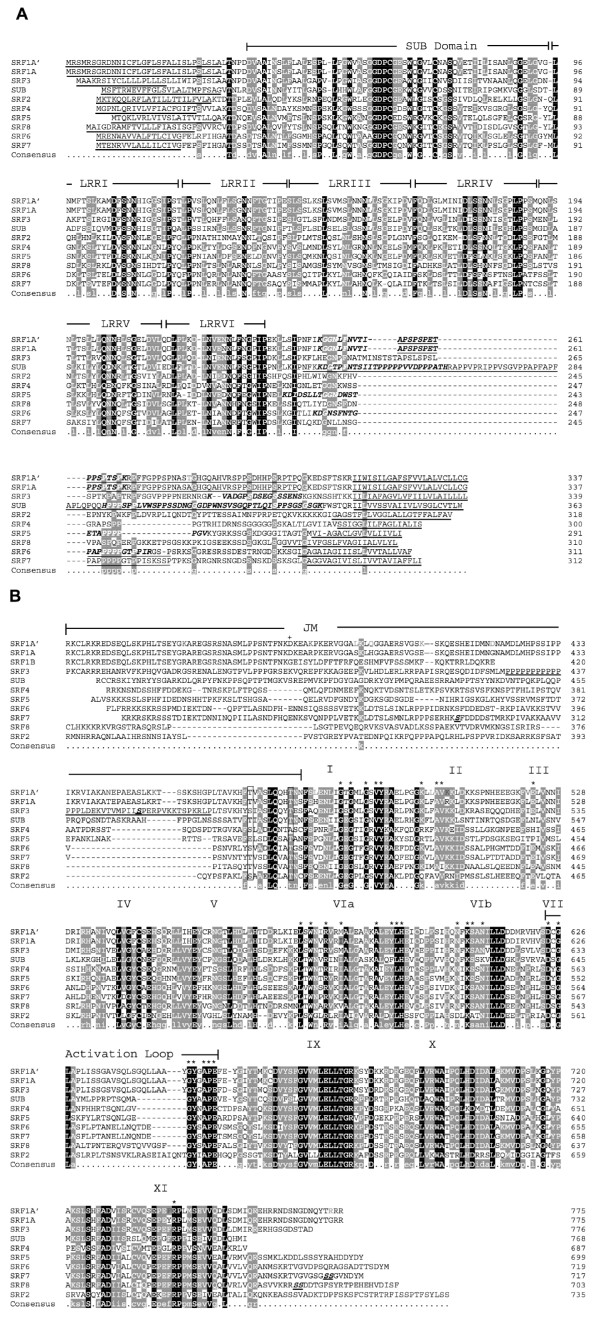
**Protein sequence alignment of SRF proteins**. SRF1A' represents the L*er *version of SRF1A. All other sequences correspond to Col. Individual protein domains are indicated above the sequences. Full conservation across the alignment is marked by black columns, partial conservation by gray columns. Red color highlights a non-conservative residue exchange. Blue color marks a conservative or semi-conservative amino acid exchange. 3a) Alignment of predicted SRF amino acid sequences up to and including the transmembrane domain. Individual protein domains are indicated above the sequences. The predicted signal peptide sequences and the transmembrane domains are underlined with thick black lines. The proline-rich regions are underlined with thin black lines. Predicted PEST sequences are kept in italic. 3b) Alignment of predicted SRF amino acid sequences from juxtra-membrane domain up to and including the C-terminus. Asteriks highlight important kinase residues as revealed by standard kinase alignments [38]. SRF1: the cross in the juxtamembrane region marks the point of deviation of the SRF1A/B sequences. The proline-rich region of SRF3 is underlined with a thick black line. The 12 kinase subdomains are indicated. The *in vivo *phosphorylation sites of SRF3 (S. Peck, pers. communication), SRF7, and SRF8 are highlighted in italics and are underlined.

**Figure 4:**
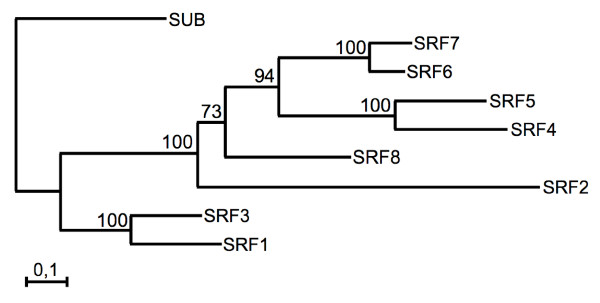
**Phylogenetic tree of *SRF *family**. A maximum likelihood tree obtained using as input the amino acid sequences of the combined SUB and kinase domains of SRF members. The branch support values are indicated.

With one distinction regarding *SRF1 *(see below), conceptual translation of the *SRF *genes suggests that they encode putative LRR-RLKs with an extra-cellular domain (ECD), a transmembrane domain (TM), an intracellular juxtamembrane domain (JM), an intracellular catalytic or kinase domain (CD), and in some cases, an extended C-terminus (Figures 2, 3A, 3B). At the amino acid level, predicted SRF members exhibit variable degrees of conservation ranging from 32.5 % identity to 77.9% identity (Table 2) and fall into distinct subclades (Figure 4) [7]. The predicted SRF proteins share an overall domain organisation. The ECDs of the LRR-V family are characterised by a stretch of 59 to 60 conserved residues representing the SUB-domain and located just N-terminal to six LRRs [29]. The role of the SUB domain is unknown. At least for SUB, however, the SUB-domain appears to be functionally relevant as the *sub-3 *allele results in an amino acid substitution at a conserved position in the SUB domain (V64M) [29].

**Table 2:** Amino acid identities among SRF proteins

	SRF2	SRF3	SRF4	SRF5	SRF6	SRF7	SRF8	SUB
SRF1	32.5	57.9	32.5	34.9	37.1	36.7	41.0	40.0
SRF2		32.8	34.4	34.9	36.0	34.8	34.8	29.6
SRF3			34.1	35.2	38.7	38.4	39.7	42.6
SRF4				55.6	43.6	44.5	40.8	32.5
SRF5					45.0	45.0	42.1	35.1
SRF6						77.9	47.5	34.2
SRF7							47.2	34.4
SRF8								34.4

The part of the ECD region that is flanked by the last LRR-repeat and the TM domain varies between the different SRF members. In case of SRF2 this region encompasses 67 residues (residues 228–294). Several SRF members feature particular regional distinctions in the central part of this domain (roughly residues 245 to 270 of SRF2). SRF1, SRF3, and SUB carry insertions in this region ranging from eight residues (SRF1) to 44 residues (SUB) while SRF4 and SRF5 each feature an identical deletion of 14 amino acids. This central part is also enriched in proline residues, particularly in SUB but also in SRF1, SRF3, SRF6 and SRF7. In the case of SRF3 an additional proline-rich domain is also located before the kinase domain.

The JM is variable among the family members. The kinase domains of the SRF proteins have the hallmarks of typical protein kinases [38] (Figure 3B). A more detailed comparison of the kinase domains, however, indicates that there are notable differences in a stretch of residues flanked by kinase subdomains II and III, a region known to be variable betweeen different protein kinases [38,39], and in the activation segment. The kinase subdomains II and III are required for the binding of ATP and the activation segment is important for substrate binding [40,41]. SUB, SRF2 and SRF8 feature unique activation segment sequences. This domain is more conserved within the SRF1/3, SRF6/7, and to some extent SRF4/5, pairs. The activation segment sequences of the individual pairs, however, are again distinct from each other and the other LRRV family activation segment sequences. This finding indicates that there may be considerable diversity in substrate recognition among family members, and therefore diversity in function. The findings also leave open possible redundant functions of members of the three more conserved pairs (but see also below).

The carboxy-termini (C-termini) of the SRF members represent another domain of diversity. SRF2 has the longest C-terminus (40 residues) while SUB and SRF4 lack such C-termini. In contrast, SRF5, the closest homolog of SRF4, features a 23 residue extension. Furthermore, only the first 13 amino acids of the SRF6 and SRF7 extensions are conserved. Interestingly, distinct serines in the C-termini of SRF7 and SRF8 (Figure 3B) are phosphorylated in an Arabidopsis suspension culture system [42].

### *SRF1 *undergoes differential splicing

During the full-length cDNA isolation experiments we noticed that *SRF1 *undergoes differential splicing resulting in two mRNA species: one lacks intron 10 (*SRF1A*), the other carries intron 10 (*SRF1B*) (Figure 1). The differential splicing event occurs in both L*er *and Col indicating that it is not related to the observed *SRF1 *L*er*/Col polymorphisms (see below). Differential splicing of *SRF1 *was observed in all tissues tested. We could not detect splicing variants for the other *SRF *members in RT-PCR experiments using primer pairs flanking the equivalent intron. Full-length *SRF1A *and *SRF1B *cDNA species were generated from RNA isolated from stage 1–12 flowers (see Methods). Conceptual translation indicates that the two SRF1 variants share the ECD and the TM but differ in their intracellular domains. Thus, *SRF1 *is likely to encode two proteins: a LRR-RLK (SRF1A) and a membrane-anchored LRR receptor-like protein (LRR-RLP) (SRF1B) that lacks most of the intracellular domain. Prominent examples of genes encoding membrane-anchored LRR-RLPs include *CLAVATA2 *(*CLV2*), *TOO MANY MOUTHS *(*TMM*) or *RPP27 *from Arabidopsis and *Cf-9 *from tomato [43-46].

### *SRF1 *is characterised by a high degree of polymorphism between the L*er *and Col accessions

**Figure 5:**
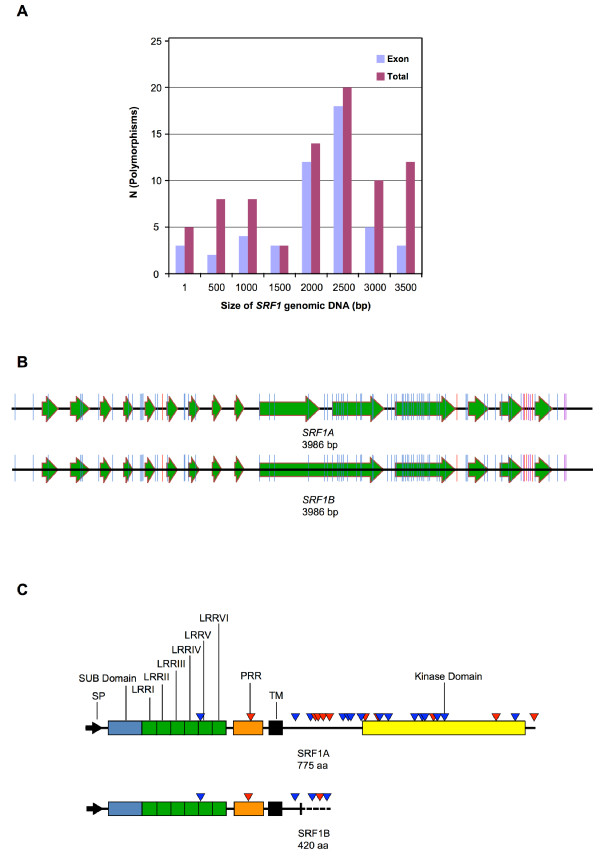
**The SRF1 L*er*/Col polymorphisms**. 5a) Frequency distribution of polymorphisms relative to the position within the *SRF1 *locus. 5b) Graphic representation of the location of the polymorphisms within *SRF1*. Large arrows indicate exons. Blue color denotes a single nucleotide polymorphism, red color marks an insertion and purple color highlights a deletion. 5c) Graphic representation of the location of the polymorphisms within the putative SRF1 proteins. The different C-terminus of SRF1B is marked by a vertical bar plus a dashed line. Polymorphisms are indicated by triangles. Red color highlights a non-conservative change, blue color a conservative or semi-conservative alteration.

The *sub *phenotype in above-ground tissues is much less prominent in the Col background [29]. An initial genetic analysis indicated the existence of a genetic modifier, located on the second chromosome and linked to the *ERECTA *(*ER*) locus (D. C. and K.S., unpublished observations). *SRF1 *is located on chromosome 2 within a short distance to *ER*. Thus, we tested whether or not there is noteworthy polymorphism in the *SRF1 *sequence when comparing the L*er *and Col accessions. We did observe an unusual amount of polymorphisms in *SRF1 *(Tables 3 and 4, Figure 5) although subsequent studies indicated that *SRF1 *is not the modifier (unpublished results, see also below). We sequenced genomic DNA of the L*er SRF1 *locus spanning nucleotides 8982429 to 8986460 (numbers as in Col) and we sequenced full-length cDNAs obtained from mRNA isolated from L*er *and Col accessions (see Materials and Methods). Within the 3.986 kb of *SRF1 *genomic sequence covering the coding sequence we observed a total of 78 L*er*/Col polymorphisms or about 20 polymorphisms per 1 kb. This is in contrast to the average number of polymorphisms between two accessions which is about 4 polymorphisms per 1 kb genomic DNA across all sequence types and somewhat lower in coding regions [47].

**Table 3:** Number of nucleotide polymorphisms in SRF1A (Col/L*er*)

Polymorphism	Exons	Introns	5'UTR	3'UTR	Total
Insertion	-	5	-	-	5
Deletion	-	3	-	2	5
SNP	47	18	2	1	68

Total	47	26	2	3	78

Ka/Ks = 1.23

**Table 4:** Location of single nucleotide polymorphisms in various SRF1A domains. Abbreviations: JM, juxtamembrane domain; LRR, leucine-rich repeat; PRR, proline-rich region; SP, signal peptide.

Polymorphism	SP	SUB Domain	LRR	PRR	JM	Kinase	C-Terminus
Synonymous	1	2	3	1	-	13	-
Non-synonymous	-	-	1	1	11*	12	1

Total	1	2	4	2	11	25	1

*Two of SNPs are located in the first and third codon position of a triplet

Ka/Ks = 0.25 (extracellular)

Ka/Ks = 1.84 (intracellular)

Abbreviations: JM, juxtamembrane domain; LRR, leucine-rich repeat; PRR, proline-rich region; SP, signal peptide.

Out of these 78 polymorphisms 68 correspond to simple nucleotide polymorphisms (SNPs) and 10 to small insertions/deletions (indels). The indels encompass 5 insertions (from 2 to 6 bp) and 5 deletions (from 1 to 6 bp). Eight indels are located in introns, and two deletions are found in the 3' UTR. Of the SNPs, 18 are located in introns, two are present in the 5' UTR, one is located in the 3' UTR, and 47 SNPs are present in exons. The other *SRF *genes do not exhibit similarly elevated levels of polymorphisms between L*er *and Col (B. E., A. F. and K. S., unpublished observations; R. Clark and D. Weigel, pers. communication).

The polymorphisms are not equally distributed along *SRF1*. At the nucleotide level about 21% of the polymorphisms are located in the region encoding the ECD whereas 79% are found in the intracellular domain. At the predicted protein level, 23 of the 25 non-synonymous residue changes map to the intracellular domain (Figure 5, Table 4), with the JM featuring 10 amino acid alterations and the kinase domain 12 changes. This finding suggests that the polymorphisms affect, in particular, SRF1A. It is unclear if the polymorphisms influence protein function. Many of those nucleotide polymorphisms may do so as they alter the predicted protein sequence. Of the nucleotide polymorphisms located in exons, 26 result in amino acid changes of which at least six result in residues with different chemical and spatial properties. However, only one such alteration, a change from proline (Col) to leucine (L*er*) at position 600 in SRF1A and situated just between kinase subdomains VIa and VIb, affects a residue strictly conserved between SRF proteins (Figure 3B). The other polymorphisms reside at positions occupied by amino acids that are not conserved or only partially conserved among the SRF proteins.

### Functional analysis of *SRF *genes

*SRF *gene function was assayed by analysing the morphology of several independently isolated T-DNA insertion lines for each *SRF *gene. In addition, we tested *srf1 srf3*, *srf4 srf5*, and *srf6 srf7 *double mutants (for a detailed description of insertion lines see Figure 1, Additional file 1, and Methods), thereby assaying the possible functional redundancy of these gene pairs. Mutant and wild-type plants were grown on soil in a greenhouse and scored in a systematic fashion for phenotypes at various developmental stages (see Methods).

**Figure 6:**
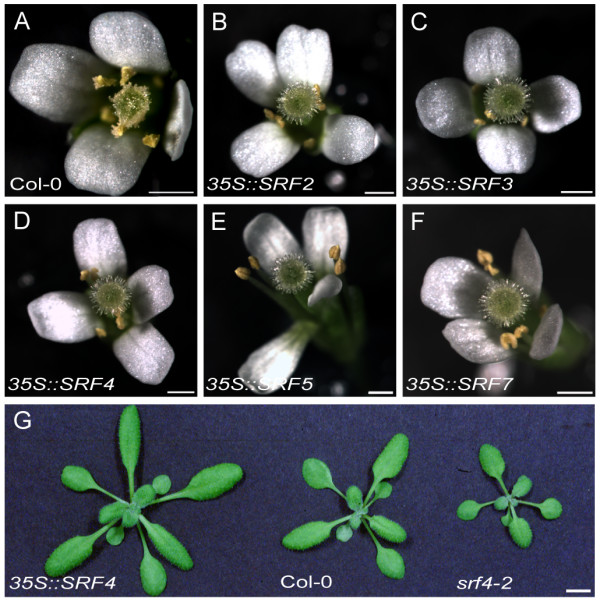
**Phenotypic effects of altering SRF expression**. 6a) An open wild-type flower. 6b-f) Flowers of same age as in 6a. Note the absence of mature pollen. 6 g) Leaves of plants with altered *SRF4 *activity. The *35S::SRF4 *(line 1–5), Col, and *srf4-3 *plants are indicated. Leaves are enlarged and reduced, respectively. Note the regular leaf shapes. Scale bars: 0.5 mm

Plants with altered *SRF4 *activity show a phenotype that indicates that *SRF4 *plays a role in the regulation of leaf size (Figure 6G, Table 5). We noticed that about 80% of homozygous mutant plants of two independent T-DNA insertion lines, *srf4-2 *and *srf4-3*, exhibited a reduction in leaf size. Leaf blade dimensions were measured using as standard the fifth rosette leaves [48] taken from different 16-days-old plants grown simultaneously and under similar conditions. Both alleles showed an approximate 20% reduction in the length and width of the leaf blade. This translates to a decrease of 40% in the surface area of the leaf blade. Interestingly, transgenic wild-type Col plants ectopically expressing *SRF4 *using the 35S promoter of cauliflower mosaic virus [49] exhibited leaves of increased size. We tested five transgenic lines with enlarged leaves and those lines exhibited elevated levels of transgene expression (not shown). Two independent homozygous *35S::SRF4 *lines were characterized further (T3 generation, lines 3–12 and 1–5, respectively). We noted a 25–30% increase in length and width of the leaf blade, translating into a 40–50% increase in the surface area of the leaf blade. Interestingly, leaf shape appeared about normal in *srf4 *and *35S::SRF4 *plants. This is also indicated by the constant length/width ratios of leaf blades across the two types of mutants and the wild type (Table 5).

**Table 5:** Blade size of fifth rosette leaves in 16 day old plants

Genotype	Length	Width	Leaf Index^a^	Perimeter	Area	n
Col-0	11.5 ± 1.8	6.8 ± 1.0	1.691	32.5 ± 5.5	63.5 ± 19.2	27
*35S::SRF4:myc.A*	13.9 ± 1.9	8.2 ± 1.0	1.695	38.0 ± 4.8	87.4 ± 23.1	25
*35S::SRF4:myc.B*	14.5 ± 1.4	8.8 ± 0.8	1.648	39.8 ± 3.8	95.3 ± 15.8	24
*srf4-2*	9.2 ± 1.4	5.5 ± 0.8	1.673	25.0 ± 4.0	39.1 ± 11.1	13
*srf4-3*	8.6 ± 1.7	5.4 ± 1.0	1.593	24.8 ± 4.5	38.9 ± 12.8	22

Values are in mm except for the area column (mm^2^). The mean ± SD is shown. The mean values of all measurements between *srf4 *mutants, *35S::SRF4 *transgenic plants and wild type are statistically significant (P < 0.001, Student's t-test).

*35S::SRF4:myc*.A and *35S::SRF4:myc*.B correspond to transgenic lines 3–12 and 1–5, respectively.

^a^Length/width ratio of leaf blade.

Taken together these findings provide genetic evidence that *SRF4 *is a direct positive regulator of leaf size. The basis of the slightly reduced penetrance in the two *srf4 *mutants is unclear. The insertions in both *srf4 *alleles reside in exons encoding part of the extracellular domain of SRF4. In particular, *srf4-2 *is predicted to carry only a very short form of the ECD (Figure 1). It is therefore unlikely that residual *SRF4 *function in *srf4-2 *accounts for the reduction in penetrance. It is also unlikely that partial compensation of *SRF4 *function by its closest relative *SRF5 *explains the reduced penetrance of the leaf size phenotype in *srf4 *mutants. The analysed mutant alleles of *SRF5*, *srf5-1 *and *srf5-2*, apparently looked normal, transgenic *35S::SRF5 *plants did not exhibit noticably bigger leaves, and *srf4-2 srf5-1 *double mutants essentially resembled *srf4-2 *single mutants (not shown). Thus, the results suggest that the reduced penetrance of the *srf4 *phenotype relates to other, as yet unknown factors.

Apart from *srf4 *mutants, all other T-DNA-induced mutants exhibited apparent wild-type morphology. We could also detect no obvious differences from wild type when light-grown mutant plants were tested for germination behavior and root growth defects on 0.5× MS agar plates, supplemented with 1% sucrose. As previously reported, the *sub *phenotype is much more pronounced in the L*er *background compared to Col [29]. As the T-DNA insertion lines are Col-derived, individual *srf *T-DNA alleles were crossed into a L*er *background (see Methods). Again, apart from the defects in *srf4 *plants, we could detect no additional phenotypes/defects in *srf *mutants. The lack of phenotypes could be in part due to the fact that the T-DNA insertions in those lines only lead to an incomplete loss of *SRF *function. We could detect transcripts in all tested T-DNA lines when using primers (Figure 1) directed to a position located 3' to the T-DNA integration site (not shown). In the case of *SRF1-5*, however, at least one line had a T-DNA insertion situated in an exon encoding part of the ECD indicating that those insertions should severly impair protein function. The situation is less clear for the insertions in *SRF6-8*. The insertion sites in *srf6-2 *and *srf6-4 *are located within 50 base pairs 5' to the start of the *SRF6 *coding region. The insertion site of *srf8-2 *resides in the 5' UTR. Therefore, the observed transcript levels could result in sufficient *SRF6 *and *SRF8 *activity. The insertion sites in *srf7-2 *and *srf7-3 *are located in introns. In both lines correctly spliced mRNA molecules could be detected by RT-PCR using primers flanking the intron carring the insertion (Figure 1) (not shown) again leaving the possibility that sufficient *SRF7 *activity is present in those T-DNA insertion lines.

To gain further indications as to the function of individual *SRF *genes transgenic plants were generated that ectopically express *SRF1A, SRF1B *and *SRF2-8 *cDNAs under the control of the 35S promoter. Again L*er *and Col wild-type backgrounds were used and transgenic plants were assayed for transgene expression (see Methods). No obvious phenotypes were detected in plants carrying *35S::SRF1B *or *35S::SRF6 *constructs. In contrast, *35S::SRF2*/*3*/*5*/*7 *plants (Col), while otherwise normal in appearance, exhibited sterility (at least 5/50 transgenic plants; Figure 6). The *35S::SRF4 *plants (Col) also showed sterility in addition to the leaf-size phenotype outlined above. Closer analysis of open flowers revealed all five different transgenic plants to carry mature anthers that in the most extreme cases failed to produce any visible pollen, while ovules appeared normal. Pollinating sterile plants with wild-type pollen resulted in normal seed production indicating that such plants were male-sterile (data not shown). In the case of different *35S::SRF4*, *35S::SRF5*, and *35S::SRF7 *lines the sterility correlated with increased transgene expression while for *35S::SRF2 *and *35S::SRF3 *lines no strict correlation could be detected (not shown). In the L*er *background only ectopic expression of *SRF3 *and *SRF4 *led to male sterility, indicating the importance of genetic background in these experiments.

The situation for *35S::SRF1A *and *35S::SRF8 *plants was more complex. We noticed that the majority of resistant T1 seedlings failed to generate rosette leaves. The seedlings featured cotyledons, stayed green for several weeks and then died (B.E., data not shown). This finding was observed in L*er *and Col backgrounds and indicates that ectopic expression of *SRF1A *and *SRF8 *may lead to seedling lethality. Conversely, viable transgenic *35S::SRF1A *and *35S::SRF8 *plants, that also expressed the transgene, were isolated. These plants showed no apparent mutant phenotype. The basis for the discrepancy is presently not understood. Unfortunately, due to the apparently normal anther development and fertility in the different *srf *T-DNA insertion lines it is presently difficult to decide whether or not the sterility exhibited by some of the *35S::SRF *transgenic plants relates to the wild-type function of the corresponding gene. A similar restriction applies to the seedling lethality.

To test if different *SRF *genes could substitute for *SUB *function we generated separate *sub-1 *plant lines, where each line was transgenic for one of the individual nine *35S::SRF *constructs. While at least 50 transgenic *sub-1 35S::SRF *T1 plants were screened per construct, no rescue of the *sub-1 *phenotype was observed. Seedling lethality, as described above, for *sub-1 35S::SRF1A *and *sub-1 35S::SRF8 *plants was again present, however, again several viable *sub-1 35S::SRF1A *and *sub-1 35S::SRF8 *plants expressing the transgene were isolated (see Methods). These plants showed a *sub-1 *phenotype. As the *sub-1 *phenotype disappears in *sub-1 35S::SUB *plants [29] our results suggest that *SRF1-8 *cannot functionally replace *SUB *in this genetic assay.

### Global gene expression analysis

**Figure 7:**
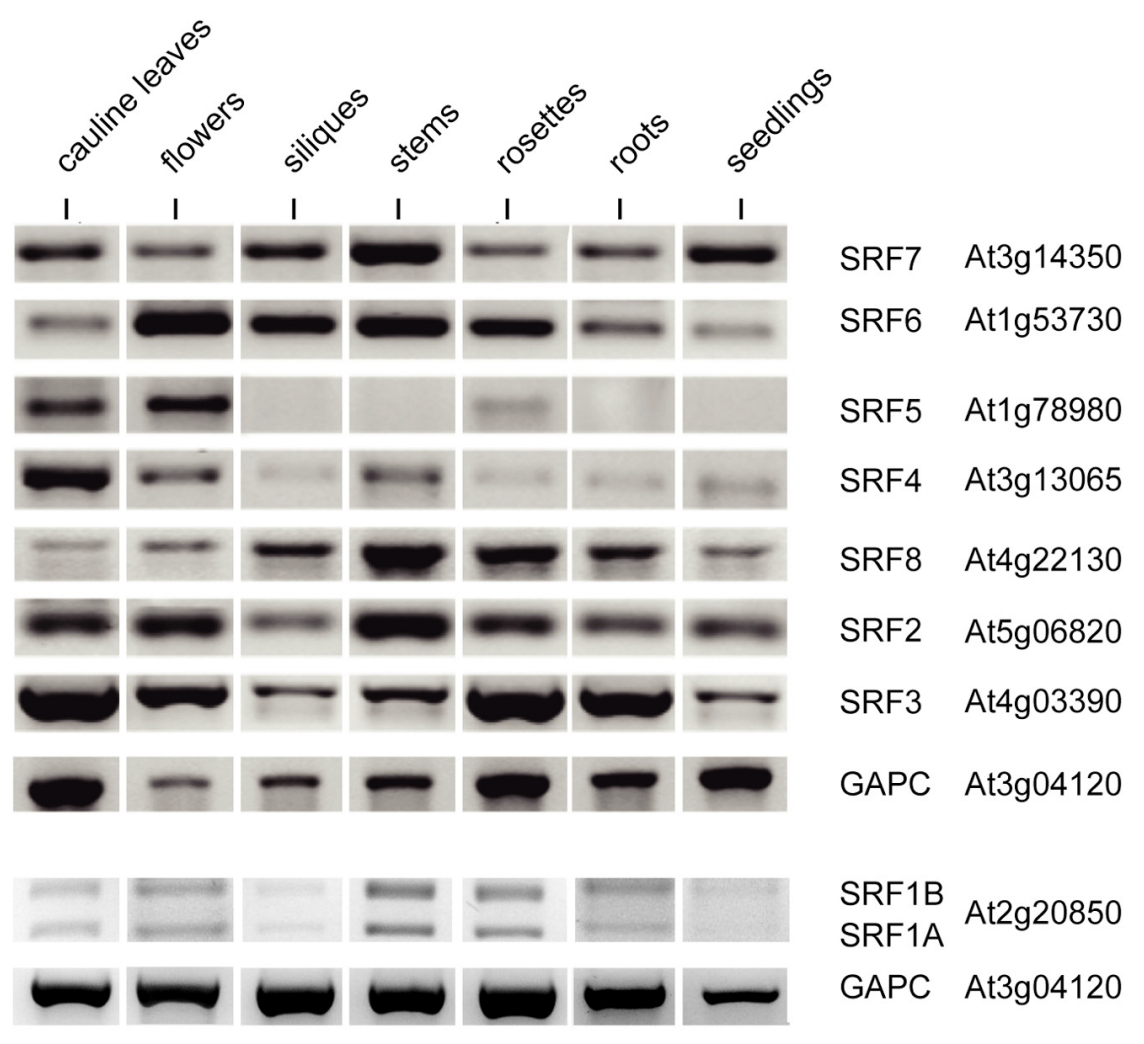
**RT-PCR-based expression profiles of *SRF1-8***. The *SRF *genes are detectable in a broad fasion, albeit at varying levels. Note the two different *SRF1*-related bands. The *SRF1*-related experiments were based on separate mRNA isolates. Therefore, a second *GAPC *control was included.

RT-PCR analysis (Figure 7) indicated that most *SRF *transcripts are present in a broad pattern. *SRF5 *expression may be the exception, as its expression levels are not easily detectable by RT-PCR, in siliques, stems, roots and seedlings. To analyse the expression profiles of *SRF *genes at a global scale, including many developmental stages and experimental conditions, we made use of a large set of GeneChip expression data publicly available at The Nottingham Arabidopsis Stock Centre (NASC) [50,51] (see Methods). The data set used comprises 1784 Affymetrix chips (ATH1 platform) and more than 100 experiments covering a wide range of tissues, developmental stages and environmental conditions. Probe sets of the ATH1 GeneChip were realigned to the Arabidopsis whole genome sequence to exclude non-unique probes. All *SRF *genes including *SUB *are described by specific probe sets (see Methods). The experimental set up did not allow a discrimination between *SRF1A *and *SRF1B*. By interrogating this dataset we targeted two objectives: to assay possible functional redundancy between *SRF *genes and to formulate hypotheses regarding the function of individual *SRF *genes.

### Correlation analysis of *SRF *transcript levels

Is there redundancy between *SRF *genes, such that an active *SRF *gene (or several *SRF *genes) could functionally replace the mutated *SRF *gene? If so, one expects at least some co-expression of the redundant genes. Therefore, we performed a global correlation analysis of *SRF *transcript levels. Pearson correlation coefficients were determined for each *SRF *pair (Table 6). To compare the *SRF *correlations against random expectation, we computed all-against-all gene pairs correlations of all genes present on the ATH1 chip (excluding self-correlations) to derive background expression similarities. Correlations were calculated as (metric) Pearson correlation coefficients. Mean and median of background distribution are r_Mean _= 0.08 and r_Median _= 0.05 and the 80%-, 95%- and 99%-quantiles are 0.51, 0.71 and 0.92, respectively. For all *SRF *pairs, global correlations are considerably below the 0.95%-quantile and, except for *SRF4 *and *SRF5*, even below the 80%-quantile (see Table 6). Thus, with the possible exception of *SRF4*/*SRF5*, global expression correlations provide no support for strong redundancies between *SRF *genes but indicate instead at least partially specific expression patterns.

**Table 6:** Pairwise Pearson correlation coefficients for the expression correlation analysis of *SRF *genes

	*SRF2*	*SRF3*	*SRF4*	*SRF5*	*SRF6*	*SRF7*	*SRF8*	*SUB*
*SRF1*	0.156	0.282	0.057	0.163	-0.06	-0.22	-0.04	0.175
*SRF2*		-0.19	0.319	0.522	-0.22	-0.21	-0.21	0.227
*SRF3*			-0.16	-0.29	0.413	0.215	0.311	0.178
*SRF4*				0.655	-0.17	-0.16	-0.15	0.046
*SRF5*					-0.23	-0.32	-0.19	-0.01
*SRF6*						0.428	0.487	0.037
*SRF7*							0.471	0.076
*SRF8*								0.295

All pairwise correlations are below the 95%-quantile of the background correlation distribution (0.71).

### Expression profiling of *SRF *genes

**Figure 8:**
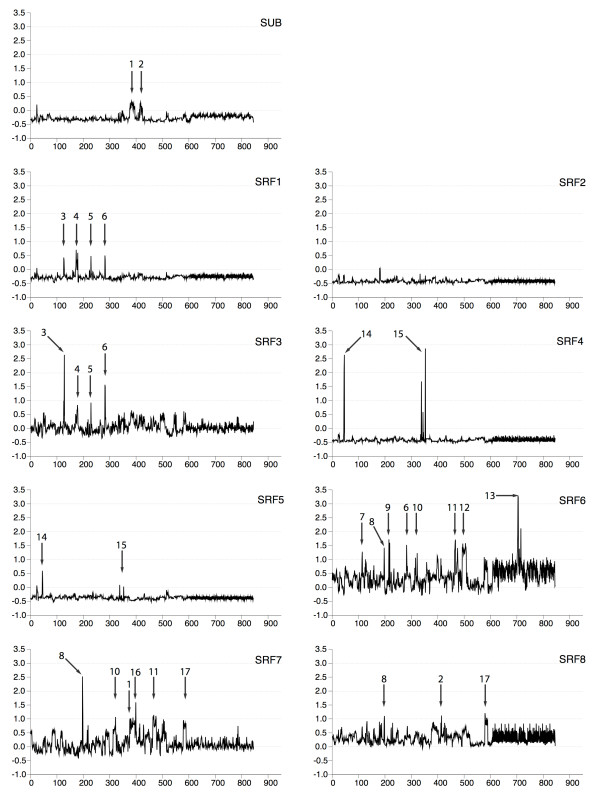
**Microarray-based expression profiles of SRF genes**. X- and Y-axis indicate (arbitrary) chip numbers and normalized expression levels, respectively. Normalised expression levels were scaled to mean 0 and variance 1. Gene names are shown at the right top corner for each profile plot. *SRF3*, *SRF6*, *SRF7 *and *SRF8 *are expressed in a large number of experiments while other *SRF*s show salient expression levels in specific experiments. Expression peaks are marked by an arrow and a number indicating the design type of the experiment (number in squared brackets refer to NASCArrays Experiment Reference Number): (1) developmental series ecotypes and mutants [No.155], (2) developmental series shoots and stems [No.153], (3) programmed cell death [No.30], (4) control of lignification [No.14], (5) tumor development [No.43], (6) pectin biosynthesis [No.27], (7) arbuscular mycorrhizal signalling [No.35], (8) AtrohbC mutant [No.42], (9) AOS burst in response to heat [No.79], (10) brassinosteroide timecourse [No.179], (11) effect of brassinosteroides in *det2 *mutants and wildtype [No.178], (12) light treatments [No.124], (13) heat stress time course [No.146], (14) transcriptional analysis of microgametogenesis [No.48], (15) developmental series flowers and pollen [No.152], (16) developmental series roots [No.151], (17) response to sulfate limitations [No.171]. Arrows point either towards a single or several conditions of one experimental design for which pronounced expression levels have been detected. The arrows represent a selection of conditions that are unique characteristics of either one *SRF *or a *SRF *subgroup.

One way to gain further leads into possible gene function is to ask whether or not a particular gene is up- or downregulated at certain developmental stages or under certain experimental conditions. To investigate expression of *SRF *genes in detail, *SRF *expression levels were analysed for each experiment in the ATH1 GeneChip dataset. Replicates were summarized by their mean. Measurements were scaled such that expression levels for each chip had a mean of 0.0 and a variance of 1.0. Expression profiles for the *SRF *genes are shown in Figure 8. *SRF *gene expression is present over a wide range of experiments and most of them show, at least for particular experiments, specific profiles. This corroborates the analysis of global expression correlations and conclusions about partly independent functionalities of *SRF *genes.

*SUB *generally has expression levels below average. It displays accented expression levels in two large developmental series. In both series, shoot apices at the bolting stage have elevated *SUB *levels. In contrast, *SRF1 *profile exhibits several distinct gene inductions or repressions in experiments investigating programmed cell death, tumor development, control of lignification and pectin biosynthesis. *SRF2 *shows an overall low and unspecific broad expression. As expected from their global correlations, *SRF4 *and *SRF5 *show the highest similarity in their profiles, with *SRF5 *having remarkably lower expression levels. Both genes appear to exhibit highly pronounced expression in mature pollen. It is possible, however, that the seemingly high levels of pollen expression in the GeneChip data set represents an artefact originating from the normalisation procedure as only 26% of the genes present on the ATH1 chip were detected in pollen samples while about 55% to 67% of the genes were detected in samples from most other tissues [51,52]. In this context it is interesting to note that *srf4 *mutants show altered leaf development but no apparent defect in pollen development or fertility. In addition, *srf5 *single mutants, and *srf4 srf5 *double mutants, show apparently normal pollen and are fertile plants. We could not confirm a notably strong expression of *SRF4 *and *SRF5 *in pollen using in situ hybridisation experiments as in our hands pollen regularly show increased background signals in such experiments (K. Pfister and K. Schneitz, unpublished observations). Although we did not perform quantitative experiments, the results from our RT-PCR analysis may indicate, however, that expression of both genes is perhaps more readily detected in developing flowers in comparison with many other tissues (Figure 7).

In contrast to the previous genes, expression profiles of *SRF3*, *SRF6*, *SRF7*, and *SRF8 *exhibit above average or high expression levels over a broad range of experiments. For these genes, we therefore describe only the most salient experiments, i.e. with the highest expression levels (arrows in Figure 8, NASC codes are given in the figure legend). The strongest expression levels of *SRF3 *are concordant to those of *SRF1*, i.e. in experiments investigating programmed cell death, pectin biosynthesis, control of lignification and tumor development. In contrast to *SRF1*, however, *SRF3 *shows mid-level expressions in many other experiments. *SRF6 *was strongly induced in plants exposed for a prolonged time (3 h) to heat stress. Considerably elevated expression levels for *SRF6 *were also observable for several light treatments, brassinosteroid treatments and pectin biosynthesis. Furthermore, strong inductions of *SRF6 *were present in experiments analyzing expression in the *Atrbohc*/*rhd2 *mutant [53] and infections with fungi inducing arbuscular mycorrhiza symbiosis. As observed for *SRF6*, both *SRF7 *and *SRF8 *showed prominent expression in experiments analyzing effects on transcription for sulfate limitations and for the *rhd2 *mutant in Arabidopsis. In addition, the expression profile of *SRF7 *showed elevated levels in experiments involving various brassinosteroid treatments.

In summary, although expression profiles showed overlapping domains between various *SRF *genes, an experiment-wide analysis of expressions corroborates our previous conclusions. That is, genetic redundancy, in the sense that at least some *SRF *genes are functionally interchangeable, is likely not a major cause for the lack of phenotypes in *srf *mutants.

### Enrichment of functional categories

Previous reports have shown that co-expressed genes have an increased likelihood to be involved in a common biological process [51,54-56]. Coexpression information can therefore be used to transfer knowledge from annotated genes to genes of unknown function. We extended our expression correlation analysis to compare individual *SRF *expression levels with the expression levels of all Arabidopsis genes included on the ATH1 chip. Gene ontology (GO) annotations for Arabidopsis genes (GO slim) were obtained from TAIR [37,57]. From the all-against-all matrix of Pearson correlations, we selected the top 100 (0.5%) correlated genes for each *SRF *gene. Overrepresentation of particular functional categories within each of these gene sets were tested by binomial probability. P-values were Bonferroni-corrected for multiple hypothesis testing. Corrected p-values of p ≤ 0.05 were considered significant. Table 7 lists the detected functional categories for the different *SRF *genes.

**Table 7:** Enrichment of functional categories within the top 100 genes correlated to *SRF *genes

SRF	GO ID	GO description	P-value
SRF3	GO:0005887	integral to plasma membrane	4.00E-04
	GO:0007030	golgi organization and biogenesis	4.08E-02
	GO:0010051	vascular tissue pattern formation) (*)	6.36E-02
	GO:0030126	COPI vesicle coat (*)	6.36E-02
SRF4	GO:0004672	protein kinase activity	3.27E-05
	GO:0004674	protein serine/threonine kinase activity	2.90E-04
	GO:0006468	protein amino acid phosphorylation	8.36E-04
	GO:0004857	enzyme inhibitor activity	8.18E-03
	GO:0030599	pectinesterase activity	3.15E-02
	GO:0016301	kinase activity	4.19E-02
SRF6	GO:0031225	anchored to membrane	1.42E-03
	GO:0009621	response to pathogenic fungi	1.78E-03
	GO:0007267	cell-cell signaling	1.23E-02
	GO:0012505	endomembrane system	1.74E-02
	GO:0048046	apoplast	3.16E-02
SRF7	GO:0009833	primary cell wall biosynthesis	1.14E-06
	GO:0005618	cell wall	2.33E-05
	GO:0016759	cellulose synthase activity	1.91E-04
	GO:0030244	cellulose biosynthesis	2.85E-04
	GO:0012505	endomembrane system	3.64E-04
	GO:0009832	cell wall biosynthesis (sensu Magnoliophyta)	6.73E-04
	GO:0016762	xyloglucan:xyloglucosyl transferase activity	5.34E-03
	GO:0008415	acyltransferase activity	9.48E-03
	GO:0004001	adenosine kinase activity	9.96E-03
	GO:0006169	adenosine salvage	9.96E-03
	GO:0031225	anchored to membrane	1.51E-02
	GO:0016798	hydrolase activity, acting on glycosyl bonds	2.18E-02
	GO:0005886	plasma membrane	2.24E-02
	GO:0007155	cell adhesion	3.57E-02
	GO:0004379	glycylpeptide N-tetradecanoyltransferase activity	3.96E-02
	GO:0009826	unidimensional cell growth	4.71E-02
SRF8	GO:0004601	peroxidase activity	1.13E-02
	GO:0016126	sterol biosynthesis	4.59E-02

Right column shows Bonferroni-corected p-values for significance of over-representation. P-values < 0.05 were considered significant.

Curiously, our analysis detected only for *SRF4*, but not for *SRF5*, a statistically significant enrichment for several functional categories. This result is somewhat surprising as both genes have a similar expression profile. Both profiles mainly differ in expression levels corresponding to their expression in mature pollen tissue while for most other experiments, both genes show basal or background expression levels. The relatively small number of pronounced or informative expression peaks for this gene pair could influence our statistical test due to many small fluctuations from noise or background expression. Therefore, in our current analysis, it is not clear whether differences between *SRF4 *and *SRF5 *truly reflect different biological roles or may be due to an insufficient resolution/power of our approach. However, we were able to detect significant enrichments for GO annotations within their correlated genes for *SRF3*, *SRF4*, and *SRF6-8*. As expected, GO terms describing kinase signaling pathways were overrepresented in several gene sets. The set of *SRF3 *was enriched in Golgi-associated processes and the set of *SRF4 *in processes regulating pectinesterase activity. *SRF6 *is potentially acting in pathways responding to fungal infections. *SRF7 *shows a strong association with proteins involved in the organization and biogenesis of the cell wall while *SRF8 *may act in sterol biosynthesis.

## Discussion

### Differential splicing at the *SRF1 *locus

We found that differential splicing of the *SRF1 *transcript potentially leads to two types of proteins: a LRR-RLK (SRF1A) and a membrane-anchored LRR-RLP (SRF1B). Genes encoding putative RLPs represent a large family in plants [8,58]. As a rule, RLKs and RLPs are encoded by separate genes in plants [8,58]. Thus, *SRF1 *is unusual as the RLK and RLP versions of SRF1 appear to be generated by differential splicing. In this respect, however, it resembles for example the *Brassica *gene encoding S-locus receptor kinase (*SRK*) from the *S*_3 _and *S*_9 _haplotypes [59,60]. Differential splicing of *SRK *in those haplotypes results in multiple transcrips, one of which encodes the SRK while another encodes a soluble protein, carrying the ECD but not the TM or intracellular domain (eSRK).

The function of most plant RLPs is unknown [8,58]. To date, RLPs are known to affect processes as diverse as meristem regulation, stomata development, self-incompatibility, or pathogen resistance [43-46,61-63]. Interestingly, RLPs such as Xa21D, CLV2, TMM, or the S-locus glycoprotein (SLG) of *Brassica*, may affect the same process regulated by a RLK with a related ECD. For example, the LRR-RLK Xa21 and the extracellular LRR-RLP Xa21D confer the same resistance spectrum to *Xanthomonas oryzae *pv *oryzae*, albeit with different strengths [63-65]. Genetic and biochemical evidence indicates that the membrane-anchored LRR-RLP CLV2 acts in the same pathway than the LRR-RLK CLV1 and that CLV2 and CLV1 form a complex [43,66,67]. In addition, there is genetic evidence that the membrane-anchored LRR-RLP TMM functions in the stomatal patterning process regulated by members of the ERECTA family of LRR-RLKs [26]. The Brassica self-incompatibility protein SLG occurs in different forms but is generally an extracellular protein with an S-domain highly homologous to the S-domain of SRK [68,69]. The exact function of SLG is still under debate [68,69], however, there is evidence that in certain *S*-haplotypes SLG is part of a protein complex that includes SRK [70,71]. SLG enhances the self-incompatibility response in some *S*-haplotypes [72] and one function of SLG may reside in the stabilisation of SRK [73].

Whether or not SRF1A and SRF1B act in the same protein complex remains to be investigated. In this context it is interesting to note that SRF1A may differ from SRF1B in its biological activity as indicated by the presence of seedling lethality exhibited by some *35S::SRF1A *plants, but not by *35S::SRF1B *plants. Thus, differential splicing at the *SRF1 *locus may result in two SRF1 protein variants with distinct biochemical and possibly biological properties.

### *SRF1 *exhibits high levels of L*er*/Col polymorphisms

A high number of polymorphisms were found when comparing *SRF1 *sequences between Col and L*er*. Several Arabidopsis genes exhibit such elevated levels of polymorphisms. These include a large number of nucleotide-binding site plus leucine-rich repeat (NBS-LRR) genes, a domain organisation characteristic of many plant resistance (*R*) genes [74-76], but also developmental regulators such as *APETALA 3 *(*AP3*), *CAULIFLOWER *(*CAL*) [77,78], or *CLV2 *[43].

Most *R *genes of the NBS-LRR class are organised in single units, clusters, and superclusters [76]. *R *genes are involved in gene-for-gene interactions [79,80]. The presence of a specific allelic variant of an *R *gene and a corresponding specific avirulence allele from the pathogen in both host and pathogen results in disease resistance. In the case of NBS-LRR-class R proteins the C-terminal LRRs are likely to be important for the specificity of R protein and avirulence protein interaction. In accordance with this view, such *R *genes are characterized by high variability in the LRR-coding regions and population genetic analysis indicates that balanced selection acts to maintain resistant and susceptible alleles [81-86]. Interestingly, the *CLV2 *polymorphisms affect mainly the N-terminal LRRs in the ECD [43], which are likely to be involved in ligand binding.

Whether or not *SRF1 *is under balancing selection remains to be seen. The large number of *SRF1 *polymorphisms is evident in a L*er*/Col comparison. *SRF1 *does not appear to diverge much from Col in several other accessions (R. Clark and D. Weigel, pers. communication). In addition, *SRF1 *is not situated in a generally polymorphic genomic region although the neighbouring locus At2g20840 also exhibits elevated levels of L*er*/Col polymorphisms (R. Clark and D. Weigel, pers. communication). At2g20840 encodes a protein annotated as a secretory carrier membrane protein (SCAMP) possibly acting in endocytosis. It should also be noted that the allele is rare and if under balancing selection, is maintained at very low frequency. The K_a_/K_s _ratio, the ratio of nonsynonymous over synonymous amino acid substitutions, may point to an influence of balancing selection. A ratio exceeding unity is compatible with balanced selection. Taking the entire SRF1 protein into account, the K_a_/K_s _ratio is 1.25 (25 non-synonymous/20 synonymous amino acid substitutions). In addition, the asynonymous changes are mainly concentrated in a particular region of SRF1. Considering the ECD and the intracellular domain (JM/CD/C-terminus) separately, one observes values of 0.29 and 1.85, respectively (Table 4). Thus, and in contrast to *CLV2*, the *SRF1 *polymorphisms mainly affect the intracellular domains of the predicted SRF1 proteins.

### *SRF4 *is required for the control of leaf size

The genetic results presented in this paper suggest that *SRF4 *is a direct positive regulator of leaf size but not leaf shape. Organ size depends on the coordination of cell proliferation and cell size [87-90]. It is poorly understood how this coordination is regulated and only few genes are known that when overexpressed cause altered organ size but do not interfere with differentiation. Such genes are postulated to be involved in the control of an organ-size checkpoint [89,91]. One key element in organ size control is the positive regulation of the duration of cell proliferation during organ development (meristematic competence of organ cells). The current evidence suggests that auxin, in an *AUXIN-RESISTANT1*-dependent fashion, upregulates transcription of the *ARGOS *gene encoding a protein of unknown biochemical function [92]. Plants that exhibit reduced or ectopic expression of *ARGOS *show reduced or enlarged aerial organs, respectively. *ARGOS *mediates its effects through *AINTEGUMENTA *(*ANT*) [92]. *ANT *encodes a member of the AP2/EREBP class of transcription factors [93-95]. Plants with altered *ARGOS *or *ANT *activities share many similarities and *ARGOS *acts as a positive regulator of *ANT *expression [92]. Plants with reduced *ANT *activity show a number of defects including a variably reduced floral organ number, narrow floral organs and reduced ovule primordium and integument outgrowth [28,93,94,96,97]. In contrast, ectopic expression of *ANT *leads to leaves and floral organs, including ovules, with increased size and normal shape [91,98]. *ANT *largely influences the final number of cells in an organ and in turn mediates its function in part through cell cycle regulators such as *CycD3;1 *[91,99].

With respect to leaves recent evidence suggests that distinct processes regulate cell proliferation and cell expansion along the longitudinal (proximal-distal) and lateral (transverse) axes, respectively [100]. In addition, the plate meristem of leaf primordia sustains two-dimensional growth of leaf blades as lamina cells divide in random directions [101]. Two genes, *ANGUSTUFOLIA3 *(*AN3*) and *GROWTH-REGULATING FACTOR5 *(*AtGRF5*), are implicated as positive regulators of cell proliferation in the plate meristem [102]. *AtGRF5 *encodes a putative transcription factor [103]. *AN3 *is identical to *GRF-INTERACTING FACTOR1 *(*AtGIF1*) [104]. *AN3*/*AtGIF1 *encodes a homolog of the animal transcriptional coactivator SYT and AN3/ATGIF1 can dimerise with ATGRF5 in yeast [102]. Plants defective in *AN3/AtGIF1 *function exhibit a reduction in leaf cells due to decreasing plate meristem activity. Loss-of-function mutations in *AtGRF5 *lead to similar, though milder effects. By contrast, ectopic expression of *AN3*/*AtGIF1 *or *AtGRF5 *results in normally shaped but larger leaves. The combined genetic and molecular data support the notion that AN3/ATGIF1 and ATGRF5 act together to promote plate meristem activity and thus leaf size [102].

The positive regulation of cell expansion is also important for leaf size and involves *ARGOS-LIKE *(*ARL*), a gene related to *ARGOS *[105]. At the organ level manipulating the levels and/or duration of *ARL *activity results in similar effects on organ size than related alterations in *ARGOS *activity. Interestingly, *ARL *is required for general cell expansion, as opposed to polar cell expansion/elongation, during organ growth. *ARL *appears to mediate BR-related signaling in general cell expansion. Thus, although *ARGOS *and *ARL *are structurally related genes that affect leaf size, they do affect different cellular processes during organogenesis. Other promoters of cell expansion and leaf size include *AtGRF1*, *AtGRF2*, and *AtGRF3*, homologs of *AtGRF5 *[103]. Single or various double-mutant combinations of single mutant alleles showed either no or only small defects in leaf growth. A *grf1 grf2 grf3 *triple null mutant, however, exhibited an approximate 32% reduction of the surface area of third leaves. *35S::AtGRF1 *or *35S::AtGRF2 *plants exhibited variably increased leaf size. A cellular analysis of the phenotypes indicated that the leaf-size defects are due to corresponding alterations in cell size [103]. In addition, several so-called *extra-small sisters *(*xs*) mutants with a defect in cell expansion, leading to reduced leaf size but normal leaf shape, have recently been isolated [106].

It remains to be seen if *SRF4 *affects cell proliferation, cell size, or a combination of both, and whether or not *SRF4 *participates in the control of the leaf size checkpoint. It is possible that *SRF4 *may affect plate meristem activity given that the leaf index (ratio of length over width) remains constant across *srf4 *mutants, wild type, and *35S::SRF4 *transgenic plants. Furthermore, *SRF4 *might be involved in cell size control as GO term enrichment analysis among genes coexpressed with *SRF4 *hints at *SRF4 *being part of a mechanism inolving pectinesterase activity. It will be interesting to determine how *SRF4 *relates to the known mechanisms regulating leaf size.

### Functions of other *SRF *genes

What is the function of the other *SRF *genes and is there redundancy due to functional overlap between individual *SRF *genes in this gene family? At present we cannot provide definitive answers to these questions. *SUB*/*SCM *affects the orientation of the cell division plane and cell number in many plant tissues [29] (Ram Kishor Yadav, Martine Batoux and K.S., unpublished observations), and influences root hair patterning [30,31]. What about the functions of the other *SRF *genes? Ectopic expression of several *SRF *genes interferes with normal development and can result in seedling lethality or male sterility due to aberrant pollen development. Regarding the analysis of the loss-of-function mutants subtle phenotypes may have been overlooked or mutants are yet to be exposed to the appropriate environmental conditions. In addition, particularly in the case of *SRF6*, *SRF7 *and *SRF8 *sufficient *SRF *activity could still be present in the analysed T-DNA insertion lines.

The *SRF *gene family may also be characterised by a level of redundancy among family members, as demonstrated in several gene families encoding RLKs [12-15,22,23,25,26,107]. Several lines of investigations, while each not conclusive in its own right, provide a tentative basis for this type of redundancy to play a subordinate role in the *SRF *gene family. Sequence differences, such as the variable, sometimes proline-rich, proximal ECD region flanked by the sixth LRR domain and the TM domain, the differences in the JM and alterations in the activation segments and the C-termini, could be interpreted that many of the predicted SRF proteins carry out separate functions. In addition, results from global pair-wise *SRF *coexpression analysis do not support the notion of redundancy among *SRF *family members. As far as sequence conservation and expression profiles are concerned the gene pairs *SRF1*/*3*, *SRF4*/*5 *and *SRF6*/*7 *may represent exceptions. However, with the exception of altered leaves in *srf4 *mutants, we did not observe obvious phenotypes in the corresponding single and double mutants. In particular, the leaves of *srf5 *looked normal. In addition, SRF4 and SRF5 feature different C-termini, the members of the three gene pairs differ in the GO term enrichments in the groups of coexpressed genes, and *SRF6 *and *SRF7 *exhibit varying expression profiles. Finally, the failure of *35S::SRF1-8 *constructs to rescue the *sub-1 *phenotype suggests that none of the tested genes can functionally replace *SUB*. Thus, the combined available evidence indicates that *SRF *genes exhibit diversity at the functional level.

What then are the hypothetical roles of *SRF *genes? Global expression profiling and the analysis of the enrichment of GO terms among genes coexpressed with *SRF *genes revealed possible functions for some of the *SRF *genes. Several *SRF *genes may be involved in cell wall biosynthesis and/or function. For example, expression profiling of *SRF1 *and *SRF3 *suggests a role for these genes in lignification and pectin biosynthesis. *SRF3 *may also have a function in the cell biology of the Golgi system and in vascular tissue pattern formation. *SRF4 *may be involved in a process requiring pectinesterase activity. *SRF6 *could play a role in the defense response against pathogenic fungi. Expression profiling of *SRF6 *also raises the possibility that this gene may be involved in stress-related processes including responses to heat and light. The *srf6 *and *srf7 *single mutants, and *srf6 srf7 *double mutants, were subjected to a set of heat-stress-related assays. However, no aberrant phenotypes were detected at the plant level (Jane Larkindale and Elizabeth Vierling, personal communication). The lack of phenotype may be due to remaining wild type *SRF6 *and *SRF7 *activities in those lines (see above). It is still possible, however, that *srf6 *or *srf7 *mutants exhibit defects that can only be detected by biochemical or cell biological assays.

The GO-term analysis of *SRF7 *raises the possibility that *SRF7 *may act in primary cell wall biosynthesis and processes requiring cellulose synthase activity. *SRF8 *may be involved in sterol biosynthesis. Perhaps this explains the seedling lethality in *35S::SRF8 *plants as sterol biosynthesis is required for embryo and seedling development [108-114]. Recently, evidence emerged indicated a link between sterol biosynthesis, cellulose synthesis and the building of a cell wall [115,116]. Future experiments will test some of these indicators regarding *SRF *function.

## Conclusion

We studied the function of the *LRR-V*/*SRF *gene family encoding putative LRR-RLKs. The genetic analysis of *SRF4 *indicates a function in the control of leaf size. With the exception of plants defective in *SUB*/*SCM *and *SRF4 *activity, *srf *single mutants, and several double-mutant combinations, did not show obvious phenotypes making it difficult to infer gene function. Results from sequence comparisons and global *SRF *coexpression analyses are compatible with the view that redundancy among members does not play a major role in this gene family. New assays for *SRF *function need to take into account novel information obtained from various sources. For example, the bioinformatic analysis of microarray expression profiles and GO term enrichments among coexpressed genes raises the possibility that some of the *SRF *genes may relate to several aspects of cell wall biology. This work provides a basis for future analysis of *SRF *function.

## Methods

### Plant work and genetics

*Arabidopsis thaliana *(L.) Heynh. var. Columbia (Col-0) and var. Landsberg (*erecta *mutant) (L*er*) were used as wild-type strains. The *sub-1 *mutant was described previously [29]. Plants were grown in a greenhouse under Philips SON-T Plus 400 Watt fluorescent bulbs on a long day cycle (16 hrs light). Dry seeds were sown on soil (Patzer Einheitserde, extra-gesiebt, Typ T, Patzer GmbH & Co. KG, Sinntal-Jossa, Germany) situated above a layer of perlite, stratified for 4 days at 4°C, and then placed in the greenhouse. The plants were kept under a lid for the next 7–8 days to increase humidity and support equal germination. T-DNA insertion lines for all *SRF *genes were obtained from various sources including SIGnAL [117], the University of Wisconsin Knockout facility [118], SAIL (Syngenta Biotechnology, Research Triangle Park, NC, USA) [119], and GABI-KAT [120]. Except for *srf7-2 *(ecotype Wassilewskija, Ws) all T-DNA insertion mutations are in the Col background. Genomic DNA of wild-type and different T-DNA insertion lines was isolated according to standard procedures. The insertion lines were screened by a PCR-based approach with corresponding T-DNA-specific and gene-specific primers.

Homozygous T-DNA insertion lines were confirmed by a different gene-specific primer set. Double mutants were generated by crossing confirmed single mutants, selfing of the F1 plants, and PCR-based progeny testing in the F2. A detailed summary is given in Additional file 1. Confirmed single T-DNA insertion mutants in Col background were also introduced into the L*er *background by crossing single mutants with L*er*, F1 selfing, and F2 progeny testing for homozygous *srf *T-DNA insertions and homozygous *er *background. Morphological inspection of single mutant (either in Col or L*er *backgrounds) and double-mutant (Col background) plants was done with plants grown in the greenhouse under regular growth conditions. The following single mutants were used in the analysis: *srf1-2*, *srf1-3*, *srf1-7*, *srf2-1*, *srf2-3*, *srf3-1*, *srf3-3*, *srf3-7*, *srf4-2*, *srf4-3*, *srf5-1*, *srf5-2*, *srf6-2*, *srf6-4*, *srf7-2*, *srf7-3 *and *srf8-2*. The following double mutants were analysed: *srf1-3 srf3-1*, *srf4-2 srf5-1*, *srf4-3 srf5-1*, *srf4-3 srf5-2*, *srf6-2 srf7-2*, *srf6-2 srf7-3*. More detailed information regarding the lines is given in Additional file 1.

To avoid crowding artefacts plants were sown at a maximum density of 5 plants per pot (7 cm × 7 cm). Following planting, individual plants were inspected for morphological alterations every two days. Assayed traits included hypocotyl length, number, size and shape of rosette leaves, trichome morphology, flowering time, stem morphology, flower morphology (including ovules and pollen), fertility, and seed size and shape. To analyse germination behavior and root development, dry seeds of wild-type, single and double-mutants were surface-sterilized, plated on 0.9% agar plates containing 0.5× Murashige and Skoog medium [121] supplemented with 1% sucrose, stratified for 4 days at 4°C, and then moved to a cell-culture room kept at 22°C and 24 hrs light. Plates were placed vertically next to vertically arranged fluorescent bulbs. Root growth was assayed after 10 days. Leaf blade measurements were done using images of dissected fifth rosette leaves that had been scanned into the computer and with the help of ImageJ software [122].

### Molecular work, DNA sequencing and cDNA isolation

For DNA and RNA work standard molecular biology techniques were used [123]. Sequences were obtained by standard cycle sequencing using an ABI 373 sequencer (PE Applied Biosystems). PCR products, genomic and cDNA clones were sequenced on both strands. Additional 5' and 3' ends were obtained through a rapid amplification of cDNA ends approach [124] using the Marathon kit (CLONTECH) and poly(A)+ RNA (Col) from flowers of stages 1–12 [125] (*SRF1A/B *to *SRF5*, *SRF7-8*) or rosette leaves (*SRF6*). The various *SRF *full-length cDNA sequences have been deposited at GenBank. For a summary see Table 1. For a list of primers see Additional file 1.

### Developmental expression profile of *SRF *genes by RT-PCR

Cauline and rosette leaves were harvested from 31 days old plants. Stage 1–12 flowers (stages according to [125]), siliques and stems were collected from 30 to 38 days old plants, roots from 14 days old plants grown on standard MS-agar plates, and seedlings were taken at 14 days. Primers were taken from the *SRF *sequences that flank the transmembrane domains. This region is very variable between the different *SRF *genes. The exception is *SRF1 *for which primers were chosen that reside in the region encoding the juxtramembrane domain. PCR conditions included the following parameters: denaturation at 94°C for 1 minute, annealing at gene-specific annealing temperature, elongation at 72°C for 1 minute, 40 cycles, final extension at 72°C for 2 minutes. The *GAPC *gene was used as control [126].

### Computer-based sequence analysis

Homology searches were done with the BLAST tool [127]. The signal peptide sequences, the proline-rich regions, the transmembrane domains, and the PEST motifs were determined using the SMART [128], PROSITE [129], PSORT [130], and PESTfind [131] websites. The kinase domains were detected through the PlantsP database [132]. The subdomain organisation of the kinase domains was inferred from published kinase alignments [38]. Sequence alignments were done with MultAlin [133,134] using the following parameters: symbol comparison table: identity, gap weight: 5, gap length weight: 0, consensus levels: high = 100% low = 60%. Phylogenetic tree analysis of the *SRF *family was performed using an amino acid sequence alignment generated by the program DAMBE [135] using a gap penalty value of 20. Using this alignment a maximum likelihood tree was generated in TREE-PUZZLE [136] with the help of the JTT model of substitution. Rate heterogeneity was estimated with the gamma distribution model with eight rate categories as described in [137]. We also tested different approaches (Baysian inference, Neighbor-joining) but the topology of the tree did not change. TREEVIEW was used to visualise the tree [138].

### Overexpression of *SRF *genes

The full length *SRF *open reading frames (ORFs) were amplified from individual full-length *SRF *cDNA clones by PCR. The *SRF *ORFs were cloned in sense orientation into a modified version of the plant transformation vector pCAMBIA2300 [139]. The modified pCAMBIA2300 vector includes a 3× myc tag and allows the generation of SRF proteins that are tagged with a 3× myc-tag at their carboxy ends (Ram Kishor Yadav and K.S., unpublished work). To this end, we cloned the PCR fragments into the 5' *Asc*I and 3' *Aat*II sites except for *SRF2 *and *SRF5*, for which we used *Apa*I at the 3' end. The various *SRF:myc *ORFs are flanked 5' by the CaMV 35S promoter and 3' by a nopaline synthase transcription termination signal. The Agrobacterium strain GV3101 was used for plant transformation [140] using the floral dip method [141]. For *35S::SRF1B:myc *and *35S::SRF2 *to *35S::SRF7:myc *at least 50 transgenic T1 *sub-1 *(in L*er *background), L*er*, and Col plants were selected on kanamycin plates (50 μg/ml) and then transferred to soil.

Transgene expression in at least 5 lines of each experiment was confirmed by RT-PCR using a gene-specific primer and a myc-tag-derived primer. In the case of *35S::SRF1A:myc*, the majority of T1 plants died at the 2-cotyledon stage, irrespective of the background (*sub-1*, L*er*, and Col). We managed, however, to isolate 11 (in *sub-1 *background), 38 (L*er*), and 29 (Col) T1 survivors. We tested 7 (*sub-1*), 8 (L*er*) and 5 (Col) *35S::SRF1A:myc *T1 lines positive in RT-PCR assays for transgene expression. No apparent modification of either the *sub-1 *or wild-type phenotype was observed in those T1 plants. The *35S::SRF8:myc *T1 plants exhibited a similar lethality at the 2-cotyledon stage. Again a few T1 plants escaped the seedling lethality (20 (*sub-1*), 58 (L*er*) and 20 (Col)). We could detect transgene expression by RT-PCR in 5 (*sub-1*), 14 (L*er*), 3 (Col) T1 plants, respectively. No apparent modification of either the *sub-1 *or wild-type phenotype was observed in those T1 plants either.

### Microscopy and art work

Pictures of plants or various plant organs were taken with an SZX12 stereo microscope from Olympus coupled to a ColorView III digital camera and using Cell^P software (Olympus Europa GmbH, Hamburg, Germany). Images were saved as TIFF files and adjusted for color and contrast using Adobe Photoshop CS2 (Adobe, San Jose, CA, USA) software on an iMac G5 computer (Apple, Cupertino, CA, USA). Composites were also generated by Adobe Photoshop CS2. Line drawings were generated using Adobe Illustrator CS2.

### Microarray analysis

Microarray data were obtained from the NASC Affywatch service (CD-ROM release as of June, 2005; [142]). To avoid complications from the comparison between different platforms, we only used measurements from the Affymetrix ATH1 GeneChip platform. Probe sets were re-calculated according to the following scheme: all oligonucleotides present on the ATH1 GeneChip of Affymetrix (sequences downloaded from [143] as of October 2004) were mapped on the whole genome sequence (MAtDB release from 24^th ^of September, 2004, [144]) and realigned against coding sequences. UTR sequences were included if the respective gene is associated with full length cDNA information. Oligonucleotides aligning to more than one gene and probes without perfect matches were excluded. For subsequent calculations, only probe sets with at least five unique probe pairs were considered. About 10% of the original probe sets led to unspecific estimates indicating the need for the re-alignments. We excluded those probe sets from our refined sets. In summary expression measurements from 21,559 genes met the quality criteria and were used for subsequent analysis.

For statistical analysis of the expression data we applied the FunDaMiner system [145]. We calculated probe set summaries for the complete dataset using MAS 5.0, dChip [146] and RMA [147]. The complete dataset was normalized by applying the LMPN method. LMPN is based on the local polynomial regression fitting method *loess *[148,149] operating on MA-scale [150]. For the correlation analysis we summarized replicates (usually 3) by the arithmetic mean. For 1784 measurements, i.e. microarray experiments, we computed the correlation matrix of all-against all probe sets. Correlations were determined as metric (Pearson) correlation coefficients. To investigate expression profiles for each *SRF *gene within in this expression data set, we determined for each experiment its mean and standard deviation (σ). Expression values in the plots are expressed as fold σ difference to the mean.

### Enrichment for GO categories

GOslim annotations for Arabidopsis were obtained from TAIR [37]. For our analysis, we considered solely GO annotations derived from the ontology describing biological processes. Gene lists were matched with the 21,559 genes analyzed in this study. GO terms annotated only once in the genome were not considered. To determine whether a set of genes correlated to a particular SRF is enriched for a specific GO term, we tested for its statistical overrepresentation within the set compared to the background (whole genome) expectation. P-values were obtained for each GO category present in the set by cumulative binomial probability:

P(k≥x)=∑k−xn(nk)pk(1−p)n−k
 MathType@MTEF@5@5@+=feaafiart1ev1aaatCvAUfKttLearuWrP9MDH5MBPbIqV92AaeXatLxBI9gBaebbnrfifHhDYfgasaacH8akY=wiFfYdH8Gipec8Eeeu0xXdbba9frFj0=OqFfea0dXdd9vqai=hGuQ8kuc9pgc9s8qqaq=dirpe0xb9q8qiLsFr0=vr0=vr0dc8meaabaqaciaacaGaaeqabaqabeGadaaakeaacqWGqbaudaqadaqaaiabdUgaRjabgwMiZkabdIha4bGaayjkaiaawMcaaiabg2da9maaqahabaWaaeWaaeaafaqabeGabaaabaGaemOBa4gabaGaem4AaSgaaaGaayjkaiaawMcaaiabdchaWnaaCaaaleqabaGaem4AaSgaaOWaaeWaaeaacqaIXaqmcqGHsislcqWGWbaCaiaawIcacaGLPaaadaahaaWcbeqaaiabd6gaUjabgkHiTiabdUgaRbaaaeaacqWGRbWAcqGHsislcqWG4baEaeaacqWGUbGBa0GaeyyeIuoaaaa@4C69@

where n is the number of all studied genes associated with a specific GO annotation, x is the number of observed genes correlated to a particular SRF and associated with this GO annotation, and p is the genomic frequency of this GO annotation, i.e. the number of genes annotated for this GO identifier divided by the number of all studied genes. P-values were Bonferroni-corrected for multiple hypothesis testing. For each SRF, the total number of tests corresponded to the number of different GO annotations of its correlated gene set.

## Authors' contributions

BE, KP, GH, DC and AF designed and performed experiments. KS conceived the study. KFXM and KS designed and coordinated the study. GH and KS wrote the paper. All authors read and approved the final manuscript.

## Supplementary Material

Additional file 1 Supplement. A Microsoft Excel file with a description of *srf *T-DNA insertion lines and a complete list of primer sequences. Click here for file
